# Intraoperative Frozen Section Biopsy for the Auxiliary Diagnosis of Transmural Intestinal Intermediate T-Cell Canine Lymphoma

**DOI:** 10.3390/vetsci12020104

**Published:** 2025-02-01

**Authors:** Felipe Gaia de Sousa, Gabrielly Milioli, José Antônio Neto, Flavia de Felice, Guilherme Chaves, Mariana Pereira, Hiasmyn Lopes, Julia Wronski, Karen Nakagaki, Suzane Beier

**Affiliations:** 1 Department of Veterinary Clinic and Surgery, Veterinary School, Federal University of Minas Gerais—UFMG, Belo Horizonte 31620-295, Minas Gerais, Brazil; gabriellybautz@ufmg.br (G.M.); suzanelb@ufmg.br (S.B.); 2 UniClinica Veterinary Clinic, Itaúna 35680-359, Minas Gerais, Brazil; josenetocruz@hotmail.com (J.A.N.); flaviadefelice@hotmail.com (F.d.F.); tiamarivet@gmail.com (M.P.); 3 Zootec Diagnostic Center, Itaúna 35680-022, Minas Gerais, Brazil; guilhermegchaves@yahoo.com.br (G.C.); mvhiasmynlopes@gmail.com (H.L.); 4 Celulavet Veterinary Diagnostic Center, Belo Horizonte 31365-000, Minas Gerais, Brazil; juliawronski@gmail.com (J.W.); karenyumi@ymail.com (K.N.)

**Keywords:** canine neoplasia, immunohistochemistry, intestinal lymphoma, T cell

## Abstract

Intestinal lymphoma is characterized by a malignant lymphoid neoplasm with multifactorial causes, variable clinical symptoms, and, in some cases, rapid progression. This article describes a case of intestinal lymphoma in a dog that had a history of diarrhea lasting two months, accompanied by episodes of hematochezia, which was treated using the CHOP 19 protocol. A seven-year-old male Golden Retriever presented to a veterinary clinic with clinical signs of diarrhea, prostration, and the presence of bloody feces, as reported by the owner. Initial examinations, including hematology, serum biochemistry, and imaging, revealed an abnormal structure in the intestinal region. Considering the circumstances, a surgical procedure was conducted with the aid of a trans-surgical frozen section biopsy. This biopsy aimed to verify the presence of the neoplasm, assess the suspected diagnosis, evaluate the viability of the surgical margins, and determine the possibility of expanding the surgical area. An enterectomy with enteroanastomosis and trans-surgical biopsy was performed, leading to an initial diagnosis of intestinal lymphoma. The tissue fragment was sent for immunohistochemistry, which ultimately confirmed the diagnosis of intestinal T-cell lymphoma. The patient underwent the CHOP protocol for 19 weeks, but during the maintenance phase, abdominal ultrasound examination findings indicated tumor recurrence. Despite being informed of the potential benefits of further chemotherapy, the owner opted not to continue treatment for the animal. Frozen section biopsy can help with diagnosis during surgery, avoiding more invasive procedures and the removal of lymph nodes, since in some cases, the systemic treatment of the disease is recommended.

## Simple Summary

Lymphomas are common tumors in dogs that originate from lymphoid tissue and can affect multiple organs and systems, often presenting as multicentric, particularly in solid lymphoid tissues such as the lymph nodes, spleen, and bone marrow. Diagnosing lymphoma can sometimes be challenging; however, a trans-surgical frozen section biopsy serves as an auxiliary method, especially when combined with cytology. Having a pathologist present during surgery can help identify the underlying cause of the problem, such as the presence of tumors in various organs. By performing a biopsy, initial suspicions can be confirmed, and it provides crucial information regarding tissue viability and the adequacy of surgical margins. If necessary, this allows for adjustments in surgical procedures. Overall, this method enhances safety during the diagnosis and management of clinical cases.

## 1. Introduction

Lymphoma is the most common hematopoietic neoplasm in dogs [1], with a frequency of 94.4% for all tumor types in Brazil [2]. It is a diverse group of neoplasms originating from the malignant transformation of lymphoreticular cells, exhibiting varying behaviors depending on the cell type involved. Lymphomas typically arise in solid lymphoid tissues and are most commonly seen in middle-aged to elderly dogs of medium to large breeds, such as Golden Retrievers, Labrador Retrievers, Boxers, German Shepherds, Setters, Rottweilers, Bernese Mountain Dogs, Dobermans, and Scottish Terriers [3,4,5]. There is no definitive evidence linking the incidence of lymphoma to a particular sex, although intact females may have a lower risk of developing the disease [6], though this requires further investigation [7]. The etiology of lymphoma is complex and multifactorial, with limited knowledge regarding specific risk factors [5,8].

Lymphoma can be classified into different subtypes based on anatomical, cytomorphological, immunophenotypic, genetic, molecular, and clinical factors [9]. However, in routine veterinary oncology, comprehensive classification of extranodal lymphomas is less commonly described compared to cutaneous lymphomas [10]. Anatomically, lymphoma is classified according to the tissues affected by the neoplastic process and can present in multicentric, intestinal (alimentary), mediastinal, cutaneous, and extranodal forms [11,12]. When lymphoma primarily affects the gastrointestinal tract, it typically presents with nonspecific and persistent signs, such as vomiting, diarrhea, lethargy, weight loss, and anorexia, with variable duration and progression [11,13]. Differential diagnosis for intestinal lymphoma include adenocarcinoma, leiomyosarcoma, leiomyoma, carcinomas, and mast cell tumors [10]. Histopathological diagnosis and immunophenotyping of lymphoma involve classifying the neoplasia based on the type of lymphocyte present in the infiltrate (T, B, or null), its proliferative activity, malignancy (low, intermediate, or high grade), and cell size (large or small) [14]. Lymphoma staging and classification are crucial for selecting the appropriate therapeutic regimen and determining the need for additional treatment options, such as surgical procedures, either alone or in combination with chemotherapy [14].

There remains a gap in the understanding of gastrointestinal lymphomas, and in some cases, diagnosis can be challenging [15]. Frozen section during surgery is a procedure that aims to aid in the diagnosis and analysis of the surgical margins of neoplasms and has been used in human medicine since 1905 [16]. This procedure is critical for the performance of accurate surgery: its outcome may guide the surgeon to continue or abort the surgical procedure [17]. In this approach, the pathologist enters the surgical room alongside the surgeon. During surgery, the samples are frozen, sectioned, and stained for examination. Based on the findings, the pathologist provides a possible diagnosis, enabling the surgeon to adjust the surgical approach accordingly. For instance, the surgeon may widen surgical margins if neoplastic cells are present, alter the method of surgery, or even decide to supplement the procedure with chemotherapy. The advantage of frozen section biopsy is that it allows for early diagnosis, expedited diagnostic–therapeutic decision-making, and a tailored clinical–surgical approach. However, confirmation through conventional histopathology remains necessary. This article aims to describe the case of intestinal lymphoma in a 7-year-old Golden Retriever with a two-month history of diarrhea and episodes of hematochezia, which was treated for 19 weeks using the CHOP protocol.

## 2. Case Presentation

An intact 7-year-old male Golden Retriever, weighing 31 kg, was referred to a veterinary clinic with a two-month history of diarrhea. For approximately two weeks prior to the veterinary evaluation, the dog had been experiencing vomiting and diarrhea, along with episodes of hematochezia. The owner had attempted treatment with ondansetron and probiotics, but the dog continued to lose weight (a 4 kg weight loss before the first evaluation) and exhibited signs of anorexia. During the clinical examination, the dog’s heart rate was 132 bpm, respiratory rate was 44 breaths per minute, and mucous membranes were pale. The rectal temperature was 38.9 °C, capillary refill time was three seconds, and dehydration was estimated at 6%. The body condition score was 6 (on a 0–9 scale), muscle condition score was 3, and there was palpable enlargement of the popliteal lymph nodes along with significant abdominal pain. Laboratory tests, including a complete blood count, serum biochemistry, and abdominal ultrasound examination, were requested. As symptomatic control, the following medications were prescribed: dipyrone (25 mg/kg, q8h, orally, for three days), omeprazole (0.7 mg/kg, q24h, orally, for three days), potemin B12 (15 mL, q12h, orally, for five days), ondansetron (0.5 mg/kg, q8h, orally, for two days), and probiotics (7.5 g, q12h, orally, for five days). The laboratory results revealed normochromic, non-regenerative microcytic anemia. Based on these findings, the initial suspicion was giardiasis. The following treatments were prescribed: Erythros (one tablet, q24h, for 45 days), Giardicid (25 mg/kg, q12h, for five days), and fenbendazole (50 mg/kg, q24h, for three days).

An abdominal ultrasound examination revealed significant thickening of the duodenal segment of the small intestine (0.94 cm), with loss of parietal stratification and moderate vascularization at this location. The abdominal ultrasound examination also showed an endoluminal large mucous content, reduced peristalsis, and possible impairment of intestinal transit. The colon had a normally thickened wall (descending colon 0.21 cm), with evident parietal stratification and consistent fecal content. There was also enlargement of abdominal lymph nodes near the affected area, but no significant changes were observed in the other evaluated segments. These abdominal ultrasound findings suggested the possibility of a neoplastic process in the intestinal wall, and exploratory laparotomy was recommended to assess whether resection of the affected intestinal segment would be feasible. Intussusception, foreign bodies, and inflammatory bowel disease were ruled out by clinical evaluation and abdominal ultrasound examination.

**Figure 1:**
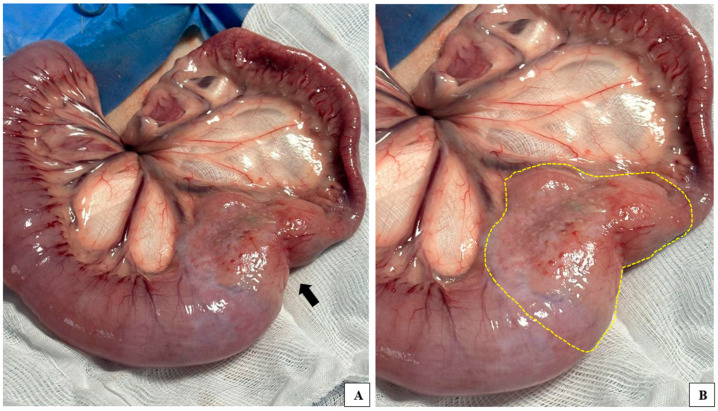
(**A**) Location of the lymphoma in the intestinal loop of the evaluated patient. Altered anatomical segments can be seen, with foci of necrosis, inflammation, and high vascularization (arrowhead). (**B**) Delimitation of the intestinal neoplastic process (yellow dashed line).

Given the worsening clinical signs and the findings on the abdominal ultrasound examination, a follow-up laboratory evaluation was performed, revealing a further decrease in hematocrit (5.5%) and a slight increase in segmented neutrophils, along with mild hypoalbuminemia. Prednisone (1 mg/kg, q24h, orally, for five days) and a soft diet were introduced before surgery to address the inflammatory response. As part of the staging process, chest radiographs (right/left latero-lateral and ventrodorsal views) were taken to rule out the presence of metastatic processes. No evidence of metastases was observed. The patient was referred for surgical intervention to remove the affected segment of the intestine, with the possibility of an enterectomy (Figure 1). A pathologist assisted during the procedure to perform a frozen section biopsy, which was intended to help with diagnosis, evaluate the surgical margins in case of neoplasia, and determine the need for regional lymph node removal.

**Figure 2:**
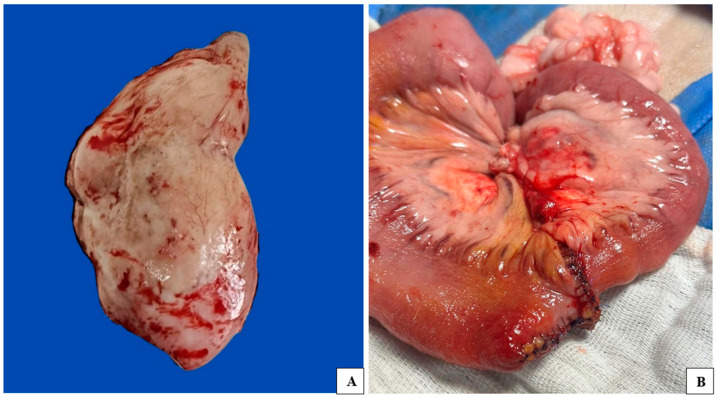
(**A**) Intestinal fragment removed after tumor excision for evaluation by frozen section biopsy, conventional histopathology, and immunohistochemistry; (**B**) Intestinal loop after enterectomy procedure associated with enteroanastomosis.

During the laparotomy, the altered intestinal loop was observed macroscopically, showing areas of necrosis, inflammation, and high vascularization (Figure 1). An enterectomy was performed, and a nodular fragment was collected for cytology and frozen section biopsy (Figure 2). Imprint cytology from the cut surface and frozen section biopsy from a 7.0 × 6.0 cm nodule in the intestinal tract were performed, along with fine-needle aspiration (FNA) cytology of a significantly enlarged mesenteric lymph node.

For cytology using FNA and imprint on the cut surface, rapid panoptic staining (Laborclin^®^, Pinhais, Paraná, Brazil), based on the Romanowsky method, was employed. In the case of frozen histopathological examination, macroscopic analysis was performed, followed by cleavage and freezing with 1,1,1,2-tetrafluoroethane (R134A), a refrigerant gas that has low flammability and low toxicity, used as a substitute for liquid nitrogen or CO_2_. Sections of approximately 5 μm were made using a portable freezing microtome, and the samples were stained with toluidine blue. These were then analyzed under a light microscope (Trinocular Biological Microscope model O500R-T, with a 5.3 MP ultrak HD OPT5 scientific capture camera from OPTICAM^®,^ São Paulo, Brazil), during the surgical procedure. Additionally, samples were collected in 10% buffered formalin for subsequent conventional histopathological analysis in the laboratory. For the conventional examination, the samples were processed, embedded in paraffin, and sectioned at a thickness of 3 μm using a manual microtome (Lupetec^®,^ São Carlos, São Paulo, Brazil), with the slides stained using hematoxylin and eosin.

**Figure 3:**
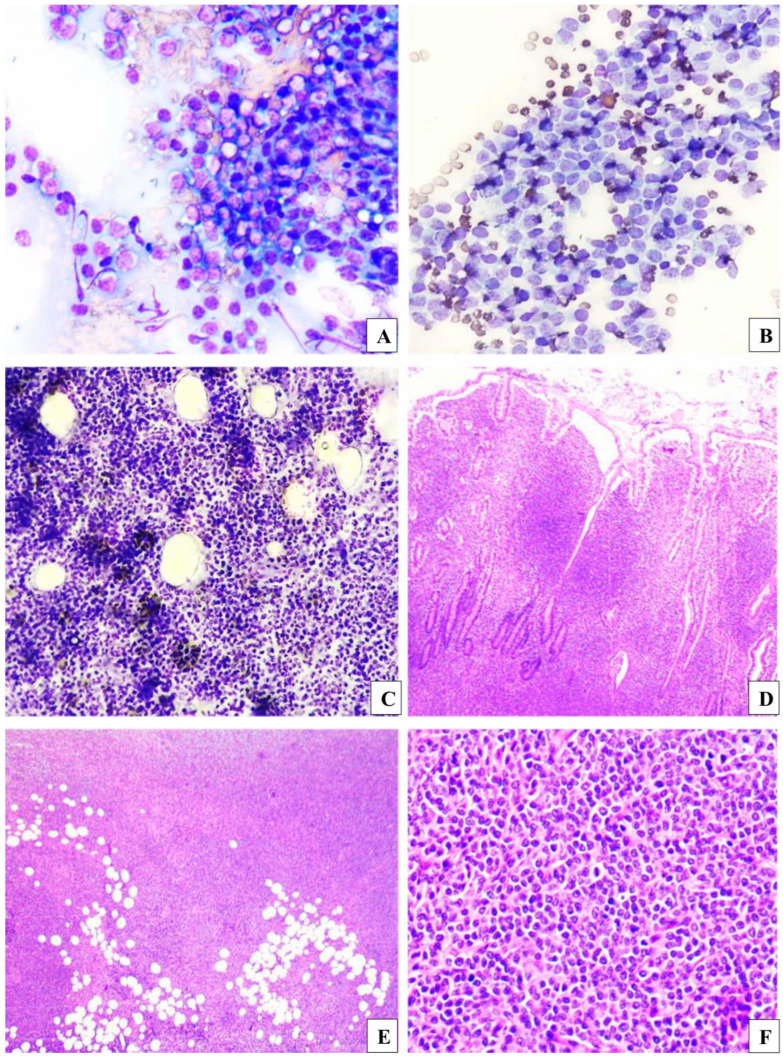
Alimentary lymphoma. (**A**,**B**) Cytology of the intestinal nodule and mesenteric lymph node showing moderate to high cellularity, composed of a monomorphic population of intermediate-sized lymphocytes, with occasionally prominent nucleoli. Stained with rapid panoptic, 400× magnification. (**C**) Frozen section revealing lymphocytes infiltrating adipose tissue in the intestinal serosa and mesenteric region. Stained with toluidine blue, 400× magnification. (**D**,**E**) Conventional histological sections demonstrating the expansion and diffuse infiltration of intestinal villi and adipocytes. Stained with H&E, 100× magnification. (**F**) Conventional histological sections showing the proliferation of a monomorphic population of intermediate lymphocytes forming a cell mantle, with occasional mitotic figures. Stained with H&E, 400× magnification.

The results from the cytology of the intestinal and mesenteric lymph nodes revealed a homogeneous population of intermediate lymphocytes. These lymphocytes had scarce, well-defined basophilic cytoplasm, which was sometimes vacuolated, and oval or occasionally cleaved nuclei with moderately prominent nucleoli. Moderate anisocytosis and anisokaryosis were observed, with approximately two mitoses present in five high-power fields (400×) (Figure 3). Histopathology of the frozen section of the intestinal fragment showed transmural infiltration of the intestinal wall by intermediate lymphocytes arranged in solid sheaths. These lymphocytes expanded the lamina propria, multifocally infiltrated the villous epithelium, and expanded the submucosa. They also dissected between muscle fibers, infiltrated the muscular and serosal layers, and extended into the mesenteric adipose tissue (Figure 3). This examination was important to guide the surgeon in the surgical approach to be taken at the time of surgery. Conventional histopathology performed later on the specimen fixed in 10% formalin confirmed the frozen section findings and intraoperative diagnosis of intermediate cell lymphoma. The sample showed 12 mitotic figures in 2.37 mm^2^ and was classified as low-grade intermediate cell intestinal lymphoma [18,19]. For more accurate diagnostic determination, the paraffin block sample was sent for immunohistochemical analysis to establish the immunophenotype. Tissue sections were mounted on slides that had been previously silanized. Antigen retrieval was performed using the moist heat method in a steam cooker for 20–30 min. Incubation with primary antibodies was carried out overnight at 4 °C. The Advance system was employed for development. Staining was performed with 3,3-diaminobenzidine, followed by counterstaining with hematoxylin. Both external and/or internal controls were used to validate the reaction. All antibodies used in the reactions were validated for cross-reactivity in canine tissue, with appropriate dilutions for this species. Immunohistochemical evaluation revealed intense lymphocyte infiltration in the intestinal mucosa, with more than 95% of the cells testing positive for CD3 (polyclonal antibody). These lymphocytes invaded the lamina propria and extended transmurally. Rare cells expressed Granzyme (polyclonal antibody), PAX5 (24/PAX-5 clone), MUM1 (BC5 clone), C-Kit (MIB-1 clone), and IBA1 (polyclonal antibody). The Ki67 proliferation marker was positive in approximately 30% of the neoplastic cells. The immunohistochemical and morphological profile supported the diagnosis of intermediate transmural intestinal T-cell lymphoma.

The animal was monitored through hematologic profiling to assess the resolution of anemia. Laboratory data from the initial diagnosis to surgical removal are shown in Table 1. The animal was under observation for two days and was prescribed meloxicam (0.1 mg/kg, q24h, orally, for three days), dipyrone (25 mg/kg, q8h, orally, for three days), and soft food. After discharge, the patient remained stable, with resolution of diarrhea and vomiting symptoms, but persistent anemia. Given this, hemoparasitosis was suspected after discussion with the owner, and an RT-PCR test was requested to rule out *Babesia* spp. and *Ehrlichia* spp. The test results were positive for both etiological agents, and treatment was initiated with doxycycline (10 mg/kg, SID, for 15 days) and imizole (5 mg/kg, with a second dose after 15 days).

Given the successful surgical excision of the tumor and the improvement in hematologic values, the patient was referred to an oncologist to begin the complementary chemotherapy protocol. One month after surgery, the patient was referred for follow-up with a veterinarian to initiate the CHOP chemotherapy protocol (vincristine, cyclophosphamide, doxorubicin, and prednisone) for 19 weeks [20,21]. Laboratory monitoring was conducted throughout the CHOP protocol. During treatment, the animal experienced anemia and episodes of neutropenia, which are expected side effects of chemotherapy. To manage these, macrogard (one tablet per 12 kg, PO) was prescribed, and filgrastim (5 µg/kg, IV) was administered as needed. The patient’s weight increased to 37 kg (a 6 kg gain since the first treatment). The protocol was completed after 19 weeks.

A follow-up abdominal ultrasound examination performed six months after completing the CHOP protocol showed no significant changes. The animal then started a maintenance protocol with vincristine (0.7 mg/m^2^ IV) every 15 days. However, two weeks after starting the maintenance protocol, an abdominal ultrasound examination revealed tumor recurrence. The owner was informed of the recurrence, along with the risks and treatment options, but chose not to authorize further treatment. The patient in this study survived for more than six months after starting the chemotherapy protocol.

## 3. Discussion

Primary gastrointestinal lymphomas are those that primarily manifest in the abdominal region, without peripheral lymphadenopathy [1]. In this report, the animal was diagnosed with intestinal lymphoma through histopathological examination and immunophenotyping. While some evidence suggests a higher incidence in Golden Retrievers, the breed is not yet considered predisposed [4,13,22,23]. Intestinal lymphoma is most commonly observed in middle-aged to elderly dogs [13,21,24,25], with the average age of affected dogs being around nine years. Clinical signs consistent with intestinal lymphoma were present in the dogs studied [13,24]. As for sexual behavior, neutered males were more affected, followed by intact males [24]. In this case, the patient was a 7-year-old intact male, which aligns with reports from other authors [24].

Common clinical signs of intestinal lymphoma include diarrhea, weight loss, vomiting, inappetence, lethargy, and apathy, with less frequent occurrence of difficulty breathing, distended abdomen, and borborygmus [13,24,26]. In this report, the patient presented with diarrhea, vomiting, and weight loss, which are typical signs [23], along with reduced serum albumin levels. Weight loss in lymphoma patients is often due to impaired nutrient absorption, resulting from increased tumor infiltration and intestinal dysfunction [24]. This fact may explain, in part, the reason that animals with lymphoma experience weight loss, since there is impairment in the nutrient absorption processes; therefore, it is expected that there will be a compromise in body gain. Muscle cachexia, abdominal distension, and pain are common clinical findings [24], though in this case, only dehydration and abdominal pain were noted, probably due to diarrhea.

Laboratory findings in dogs with intestinal lymphoma are not always remarkable. In one study, approximately 43% of affected dogs had normal blood counts [24]. Hematocrit values (<40%) were reduced in five animals, and there was high number of platelets >400,000 cells/mL only in two dogs, as well as monocytosis and neutrophilia in approximately 14 and 29% of dogs, respectively [24]. The findings of the present report are in part consistent with those described by the other authors [11,13,24]. In our patient, mild changes were noted in liver enzymes (ALT), total proteins, and albumin levels, consistent with findings in other lymphoma cases, which often show hypoproteinemia due to reduced albumin and globulin, as well as altered serum calcium, urea, and creatinine [24]. Following chemotherapy, a decrease in lymphocyte counts is typically seen [11], but this was not observed in the present case.

Definitive diagnosis of lymphoma is made through histopathology and/or immunohistochemistry, which helps characterize the phenotype of the neoplasm. In this report, the lymphoma involved the intestines and associated mesenteric lymph nodes, which were affected in 48% of dogs in a similar study [13]. Intestinal lymphoma can be confined to the gastrointestinal tract or also affect other extranodal sites. Differential diagnoses like intussusception, foreign bodies, and inflammatory bowel disease were ruled out in this case.

Ultrasonographic evaluation of intestinal segments can reveal changes indicative of intestinal lymphoma [1]. In a study, nearly 80% (n = 65/84) of animals diagnosed with lymphoma underwent ultrasound, with the most common findings involving the intestine, followed by the lymph nodes and liver [13]. In the present case, ultrasound revealed signs of intestinal abnormalities, including segmental thickening, alterations in stratification, disrupted peristalsis, impaired transit, and moderate vascularization. These findings are consistent with those reported by other authors [13,23,24].

Cytological examination is a valuable diagnostic tool that helps characterize cell types through aspiration, aiding in early diagnosis. It can provide useful information about the condition and, in some cases, is indicative of patient survival [27]. In a study [13], lymphoma was diagnosed through cytological or histopathological evaluation of tissue samples in 33% of dogs, with a positive tumor confirmation in 31%. Diagnostic sample collection can be performed via endoscopy or exploratory laparotomy [paulin 2008], and cytology can also help predict prognosis [27]. In the present case, cytological examination revealed a monomorphic population of lymphocytes, predominantly intermediate in size, suggesting intermediate cell lymphoma. These findings are consistent with those reported in other studies [15,27].

Histopathological evaluation is a diagnostic method that allows for the observation and characterization of the cells within a tissue sample, including the assessment of malignancy, staging, and cellular invasion. In this case, intraoperative pathological consultation played a critical role in determining the diagnosis and prognosis of the lesion, enabling faster initiation of treatment while avoiding more invasive surgeries, as systemic treatment is essential for lymphoma. A frozen section biopsy is a diagnostic technique that allows for the analysis of tissue or organic fragments during the surgical procedure, providing a preliminary diagnosis [28]. In this case, the diagnosis made by the pathologist at the time of surgery avoided the unnecessary removal of lymph nodes or other affected tissues, in addition to conservative intestinal surgery, without removing a large segment of the intestine, since the treatment is considered systemic and non-surgical. The removal of a segment of the intestine was only because that region was obstructing the passage of contents. If the diagnosis at the time of surgery had been of another neoplasm such as sarcoma, the conduct would have been different, aiming at the removal of the entire neoplasm as treatment. The definitive diagnosis was confirmed by histopathological examination after the frozen section biopsy results [28]. The tissue sample exhibited characteristics consistent with intestinal lymphoma, showing transmural infiltration by neoplastic lymphocytes extending from the villi to the serosa and adipose tissue of the mesentery. These findings are in line with other reports [15,23], particularly in relation to small- and large-cell lymphomas; although the mitotic count indicates a low-grade lymphoma, the predominance of intermediate-sized lymphocytes may indicate a transition from low-grade to high-grade lymphoma.

Most animals affected by lymphoma present T-cell lymphomas, which exhibit high immunostaining for CD3 in both the cytoplasm and membrane [12,13,29]. T-cell lymphomas are associated with a more deleterious prognosis compared to B-cell lymphomas, as noted in the present report [12,13,29]. Immunophenotyping is currently considered a valuable tool for classifying lymphomas, with histopathology followed by immunohistochemistry of the biopsy samples being the gold standard method [30]. Identifying the immunophenotype enables better assessment and informed decisions regarding therapeutic options, particularly in terms of the lymphoma’s behavior, chemotherapy response, and prognosis [31]. Approximately 10–38% of lymphomas are classified as T-cell immunophenotype, with the remainder categorized as B-cell or null phenotypes [12]. In the present report, immunohistochemical analysis revealed that over 95% of cells were positive for CD3, with 30% of neoplastic cells staining positive for Ki67. There was also a reduction in Granzyme, PAX5, MUM1, C-Kit, and IBA1 expression. These immunohistochemical findings align with those reported by other authors [23,24,30,32]. In a previous study [13], immunophenotyping was performed in 22 dogs, of which 91% (n = 20/22) were diagnosed with T-cell lymphoma.

Several therapeutic options are available for tumor control, ranging from isolated neoplastic resections to combination approaches, such as surgical removal followed by chemotherapy. Chemotherapy, often in combination with multiple pharmacological agents, is the most widely used treatment modality. It can be administered with a single agent or as part of a combination therapy. Common chemotherapy protocols include L-asparaginase with CHOP (L-CHOP), monoclonal antibody therapy, and others such as MOPP (mechlorethamine, vincristine, procarbazine, and prednisone), LOPP (lomustine, vincristine, procarbazine, and prednisolone), CEOP (cyclophosphamide, vincristine, epirubicin, and prednisolone), LEOP (cyclophosphamide, vincristine, epirubicin, and lomustine), and VELCAP-TSC (vincristine, L-asparaginase, cyclophosphamide, lomustine, doxorubicin, prednisolone, procarbazine, and mechlorethamine) [32]. In one study [13], 58% (n = 49/84) of affected dogs received chemotherapy, 29% (n = 24/84) underwent surgery in combination with chemotherapy, and only 5% (n = 4/84) were treated solely by tumor removal. As with any surgical procedure, both trans- and post-surgical complications can arise, so it is essential to inform the owner about the potential risks. In the same study of 84 dogs with lymphoma, 31 dogs underwent enterectomy and enteroanastomosis as part of neoplastic control. Among these, 10% (n = 3/31) experienced complications, including septic peritonitis and fulminant pancreatitis. In the present report, the patient underwent enterectomy with enteroanastomosis without any intraoperative or postoperative complications. The patient recovered well and resumed eating easily and was subsequently placed on a chemotherapy protocol.

The patient was referred to the oncologist to initiate the adjuvant chemotherapy protocol, which is considered one of the most widely accepted treatments for intestinal lymphomas [20,21,30]. The CHOP protocol, which combines vincristine, cyclophosphamide, doxorubicin, and prednisone, remains one of the most commonly used chemotherapy regimens [21]. Although new therapeutic strategies are emerging, the CHOP protocol remains the first-line treatment [20]. The duration of the protocol typically ranges from 12 to 25 weeks [20]. In one study, the effectiveness of a 12-week versus a 19-week CHOP protocol was compared [21]. The 19-week protocol was shown to provide a longer period without relapse and a more extended remission compared to the 12-week version. As a result, the 19-week CHOP protocol was chosen for this case.

In the present report, during the 19-week CHOP protocol, the animal exhibited signs of anemia, leukopenia due to neutropenia, and occasional lymphopenia. Anemia and neutropenia are commonly observed during the 19-week CHOP treatment [21,25]. The severity of anemia was even lower in the 19-week CHOP protocol compared to the 12-week version [21]. Anemia is considered a prognostic indicator of survival in lymphoma cases [33]. Various chemotherapy protocols can be employed for treatment [13], and anemia may also be associated with paraneoplastic syndrome, a condition often observed in multicentric lymphomas [34]. Additionally, factors such as anemia, the T-cell immunophenotype, and elevated liver enzymes, like alanine aminotransferase, are linked to a reduction in overall survival time [21]. In cases of resistant lymphoma, protocols such as MOPP or MVPP may be considered, with the primary difference being the replacement of vincristine with vinblastine [35].

Furthermore, the survival time following diagnosis in these animals is generally limited, with death occurring within weeks to months [13]. In the present case, after undergoing surgical enterectomy to remove the neoplastic fragment, the patient began the chemotherapy protocol and survived for more than six months. However, after completing the CHOP chemotherapy protocol and entering the maintenance phase, tumor recurrence was observed. In a previous study [21], approximately 89.5% (n = 17/19) of the animals achieved complete remission. Following tumor excision and during the chemotherapy protocol, the animal remained free of tumor recurrence for approximately 240 days. The average overall survival in the study was 351 days, with a median progression-free survival of 245 days [21]. For animals with T-cell lymphoma, the median overall survival was longer with the 19-week CHOP protocol.

## 4. Conclusions

In conclusion, lymphomas can manifest clinically in various ways, influenced by factors such as location, cellular immunophenotyping, multicentric capabilities, and distinct progression patterns. In the present report, the patient was diagnosed with intestinal T-cell lymphoma and underwent a chemotherapy protocol, but experienced tumor progression and malignancy, leading to relapse shortly after completing chemotherapy. The use of a trans-surgical frozen section biopsy was useful in aiding the diagnostic determination of the tumor in this patient, providing valuable cellular characteristics of the affected area. Although diagnosis by frozen sections is auxiliary, it allowed diagnosis during surgery, assisting in surgical conduct and avoiding invasive procedures not indicated for this type of neoplasm that requires systemic treatment. Trans-surgical biopsy is a valuable tool and should be recommended for diagnosing organic, potentially tumoral lesions and to inform personalized therapeutic decision-making for each patient.

## Figures and Tables

**Table 1:** Results observed in the patient’s laboratory tests throughout the clinical care until the surgical procedure and resolution.

Year 2023	19 July	02 August	07 August	10 August	05 September	19 September	Reference for Adult Dogs
**Erythrogram**							
Hematocrit (%)	**35.30**	**29.80**	-	**26.40**	**35.40**	**34.90**	37.0–55.0
Hemoglobin (g/dL)	12.30	**9.60**	-	**8.60**	12.30	**11.40**	12.0–18.0
RBC (×10^6^/mL)	6.10	**5.00**	-	**4.60**	**5.40**	5.50	5.5–8.5
MCM (fL)	**58.30**	**59.40**	-	**57.80**	65.60	63.50	60.0–77.0
MCHC (g/dL)	34.80	32.20	-	32.60	34.70	32.70	32.0–36.0
MCH (pg)	20.30	**19.10**	-	**18.80**	22.80	20.70	19.5–24.5
Erythroblast (cells/100 WBC)	0	0	-	0	0	0	
Platelets (×10^6^ cells/mL)	331	349	-	274	310	380	175–500
**Leukogram**							
Total WBC (cells/mL)	9900	16,100	-	14,000	8900	11,700	6000–17,000
Rods (cells/mL)	0	0	-	0	0	0	0–300
Segmented (cells/mL)	7425	**13,685**	-	11,340	6319	8892	3000–11,500
Eosinophils (cells/mL)	198	322	-	840	712	234	100–1250
Lymphocytes (cells/mL)	1881	**16,100**	-	1400	1424	1989	1000–4800
Reactive lymphocytes (cells/mL)	0	0	-	0	0	0	
Monocytes (cells/mL)	396	483	-	420	445	585	149–1350
Basophils (cells/mL)	0	0	-	0	0	0	rare
**Serum biochemistry**							
Urea (mg/dL)	28	29	-	31	-	-	20–56
Creatinine (mg/dL)	1.1	0.8	-	1.2	-	-	0.5–1.5
A.L.T. (U/L)	40	**19**	-	51	-	-	21–102
Total protein (g/dL)	6.2	**5.2**	-	4.6	-	-	5.4–7.1
Albumin (g/dL)	2.4	**2.0**	-	**2.0**	-	-	2.3–3.1
Globulin (g/dL)	3.8	3.2	-	**2.6**	-	-	2.7–4.4
A/G ratio	0.6	0.6	-	0.8	-	-	0.6–1.1
Lipase (U/L)	-	-	181	-	-	-	25–750

Highlighted values were those considered to be different from the reference values for the species.

## Data Availability

The raw data supporting the conclusions of this article will be made available by the authors on request.
